# Adding Life to Years: Adopting Age-Friendly Public Health in Washington State

**DOI:** 10.1093/geroni/igaa057.336

**Published:** 2020-12-16

**Authors:** Carolyn Ham, Marci Getz, Janna Bardi

**Affiliations:** Washington State Department of Health, Olympia, Washington, United States

## Abstract

Age-Friendly Public Health is an emerging concept and practice aiming to equip the public health infrastructure to more readily address aging issues. In 2019, the Washington State Department of Health (WA DOH), began operationalizing Age-Friendly Public Health within the agency. Work entailed bringing together a cross-agency workgroup on healthy aging and drawing on expertise from agency programs including environmental health, immunizations, falls prevention, emergency preparedness, chronic disease and many others. The workgroup conducted an internal assessment of ‘touch points’ regarding aging work within WA DOH. The Life Course Perspective was used as a theoretical framework to categorize current efforts, and existing research on social determinants of health in aging were used to identify potential areas for future impact. In addition, the Healthy Brain Initiative (HBI) Road Map for public health was used to identify current and potential approaches to addressing Alzheimer’s Disease and Related Dementias. This workgroup inventoried WA DOH’s current efforts that align with the HBI agenda, and identified 88 opportunities to promote healthy aging and brain health. This work contributes to the emerging field of Age-Friendly Public Health by demonstrating strengths and challenges of advancing healthy aging through state public health agencies.

